# Waiting for a hand: saccadic reaction time increases in proportion to hand reaction time when reaching under a visuomotor reversal

**DOI:** 10.3389/fnhum.2013.00319

**Published:** 2013-07-01

**Authors:** Irene T. Armstrong, Melissa Judson, Douglas P. Munoz, Roland S. Johansson, J. Randall Flanagan

**Affiliations:** ^1^Centre for Neuroscience Studies, Queen's UniversityKingston, ON, Canada; ^2^Department of Psychology, Queen's UniversityKingston, ON, Canada; ^3^Department of Biomedical and Molecular Sciences, Queen's UniversityKingston, ON, Canada; ^4^Section for Physiology, Department of Integrative Medical Biology, Umeå UniversityUmeå, Sweden

**Keywords:** eye-hand coordination, saccadic reaction time, reaching movements, motor adaptation, humans

## Abstract

Although eye movement onset typically precedes hand movement onset when reaching to targets presented in peripheral vision, arm motor commands appear to be issued at around the same time, and possibly in advance, of eye motor commands. A fundamental question, therefore, is whether eye movement initiation is linked or yoked to hand movement. We addressed this issue by having participants reach to targets after adapting to a visuomotor reversal (or 180° rotation) between the position of the unseen hand and the position of a cursor controlled by the hand. We asked whether this reversal, which we expected to increase hand reaction time (HRT), would also increase saccadic reaction time (SRT). As predicted, when moving the cursor to targets under the reversal, HRT increased in all participants. SRT also increased in all but one participant, even though the task for the eyes—shifting gaze to the target—was unaltered by the reversal of hand position feedback. Moreover, the effects of the reversal on SRT and HRT were positively correlated across participants; those who exhibited the greatest increases in HRT also showed the greatest increases in SRT. These results indicate that the mechanisms underlying the initiation of eye and hand movements are linked. In particular, the results suggest that the initiation of an eye movement to a manual target depends, at least in part, on the specification of hand movement.

## Introduction

Hand movements to visual targets are typically accompanied by saccadic eye movements that bring gaze to the target ahead of the hand. When reaching to targets presented in peripheral vision, the eyes generally begin moving before the hand (Prablanc et al., 1979; Biguer et al., 1982; Jeannerod, 1988; Land et al., 1999; Johansson et al., 2001; but see Bekkering et al., 1995). However, much of the delay in hand movement onset, relative to eye movement onset, can be attributed to the greater inertia of the arm. Indeed, a recent study demonstrates that the motor commands underlying coordinated eye and hand movements appear to be issued in close temporal proximity and that commands for hand movement may even precede those for eye movement (Biguer et al., 1982; Gribble et al., 2002; Sailer et al., 2005). Given this timing, an important question is whether the mechanism underlying eye movement initiation is dependent on processes responsible for the planning and control of hand movement.

Several lines of evidence indicate that hand movement can influence saccadic initiation. For example, saccadic reaction time (SRT) is greater when eye movement is accompanied by hand movement compared to when the eyes move alone (Mather and Fisk, 1985; Bekkering et al., 1995) and SRT and hand reaction time (HRT) both increase when reaching to targets in contralateral versus ipsilateral space (Fisk and Goodale, 1985). In addition, people appear to be unable to shift their gaze away from the current reach target (toward a new gaze target), until the hand completes the reach (Neggers and Bekkering, 2000, 2001).

We investigated hand-eye coupling using a task in which participants moved a cursor, controlled by the unseen hand, to reach targets located at varying distances to the left or right of a central start position that also served as the initial fixation point. The targets and cursor were presented in the plane of hand motion. We sought to manipulate HRT by adapting participants to a visuomotor reversal (180° rotation) between the hand and the position of the cursor. Under the reversal, a hand movement to the right resulted in a cursor movement to the left and vice versa. We expected HRT to increase under this visuomotor reversal (Fitts and Deininger, 1954; Fernandez-Ruiz et al., 2011). Under the hypothesis that processes involved in the programming of hand movement influences saccade initiation, we predicted that the reversal would also result in an increase in SRT despite that fact that the task for the eyes—shifting gaze to the target—is ostensibly unchanged.

Hand movement may not only influence the initiation but also the execution of saccades. Recent studies have shown that saccadic velocity increases when saccades are accompanied by coordinated hand movements to the same target (Epelboim et al., 1995; Snyder et al., 2002) but not when the hand is directed to a second target located in the opposite direction of the saccadic target (Snyder et al., 2002). The latter result could arise because eye and hand movements are aimed in different directions or because they have different spatial goals. By examining eye only and eye plus hand movements under the visuomotor reversal, we can address this question. If hand movement increases the velocity of accompanying saccades under the visuomotor reversal, we could conclude that it does so because of a shared spatial goal independent of hand movement direction *per se*.

## Method

All procedures were approved by the Queen's University human research ethics board and were in compliance with the Helsinki declaration.

### Participants

Six university undergraduates (5 women and 1 man) participated in this study after giving informed consent. All were right handed and all had normal or corrected-to-normal vision. All participants completed two experiments.

### Apparatus

**Figure 1:**
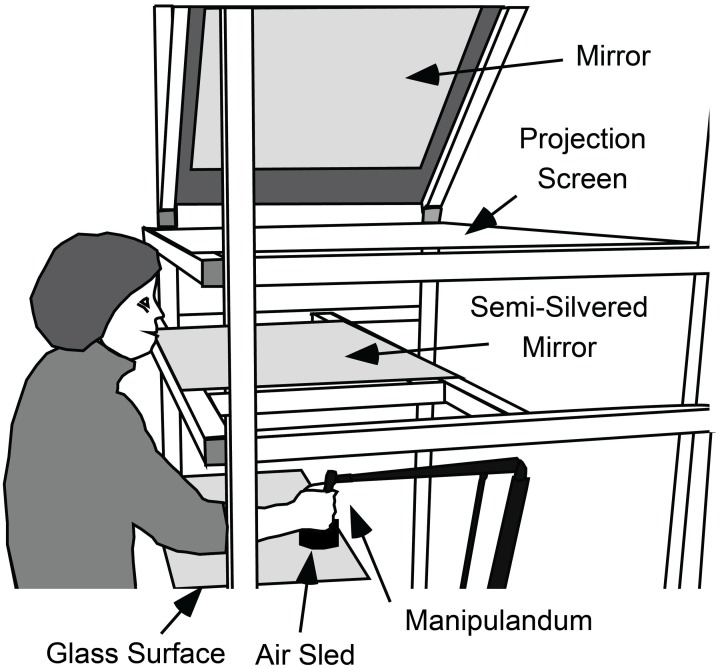
**Apparatus used to measure gaze and hand movements and to present visual feedback about targets and cursor position.** While seated, participants used their right hand to grasp the handle of a light-weight manipulandum that measured the position of the hand in three-dimensions. The handle of the manipulandum was supported by an “air-sled” that rode across a horizontal glass surface on a cushion of air. With this support, the hand was effectively constrained to move in a horizontal plane. A video projector (not shown), above and to the right of the participant, projected targets, and cursor onto a projection screen via a 45° mirror. Participants viewed the cursor targets in a semi-silvered mirror located halfway between the projection screen and the plane of hand movement and could not see their hand. Thus, the targets and cursor appeared in the same plane as the hand.

Participants grasped the handle of a light-weight manipulandum (Phantom Haptic Interface 3.0, Sensable Technologies, Inc., Cambridge, MA) that measured the position of their dominant hand in three dimensions at 1000 Hz. The handle was constrained to move in a horizontal plane (see Figure 1). A visual projection system that prevented vision of the hand and arm was used to display a start marker, visual targets, and a cursor controlled by the hand (all 1 cm diameter circles) in a horizontal plane located at the top of the handle. The start marker was located 32 cm below and 33 cm in front of the left eye and was thus about 46 cm from the eye.

An infrared video-based eye-tracking device (RK-726PCI pupil/corneal tracking system, ISCAN Inc., Burlington, MA) recorded the gaze position of the left eye in the horizontal plane of the targets at 240 Hz. To obtain accurate recordings, the head was stabilized using a forehead rest and a small bite bar. To calibrate gaze, we first used ISCAN's 5 point calibration procedure and then performed an additional 25 point calibration (Johansson et al., 2001). In both cases, the calibration points (5 or 25) were projected onto the horizontal plane of the targets and distributed such that the outer rectangle formed by the points enclosed the locations of the targets used in the experiment. We calibrated a plane rather than just a line (alone which the targets were presented) so that we could measure any gaze errors in any direction in the plane. The spatial resolution of gaze in the horizontal plane, defined as the average standard deviation of all calibrations, was 0.31 cm. This translated into an error of 0.36° visual angle when gaze is located at the start position. Gaze was calibrated before the experiment began and the calibration was checked throughout the experiment. Additional calibrations were performed if necessary; however, a single calibration was usually adequate.

### Design and procedure

All participants completed two experiments. All completed Experiment 1 first and completed Experiment 2, on average, 2 weeks later.

#### Experiment 1

A trial began when the centrally located start marker appeared on the display. Participants were required to fixate and, in trials involving hand movements, move the cursor to this marker. An eccentric target appeared once the participant's gaze and cursor were within 2 and 0.5 cm, respectively, of the start marker position for half a second. Participants were asked to move their gaze or, in hand movement trials, the cursor to the target as quickly and accurately as possible and then maintain gaze or the cursor at the target until it disappeared 2 s after target presentation. In trials in which participants were required to move the cursor to the target, no explicit instruction was given regarding eye movement; however, participants always shifted their gaze to the target and held their gaze at the target while the cursor remained at the target. The target appeared at one of three eccentricities (5, 10, and 15 cm; 6.2, 12.3, and 18.1° of visual angle) to the left and right of the start marker.

**Figure 2:**
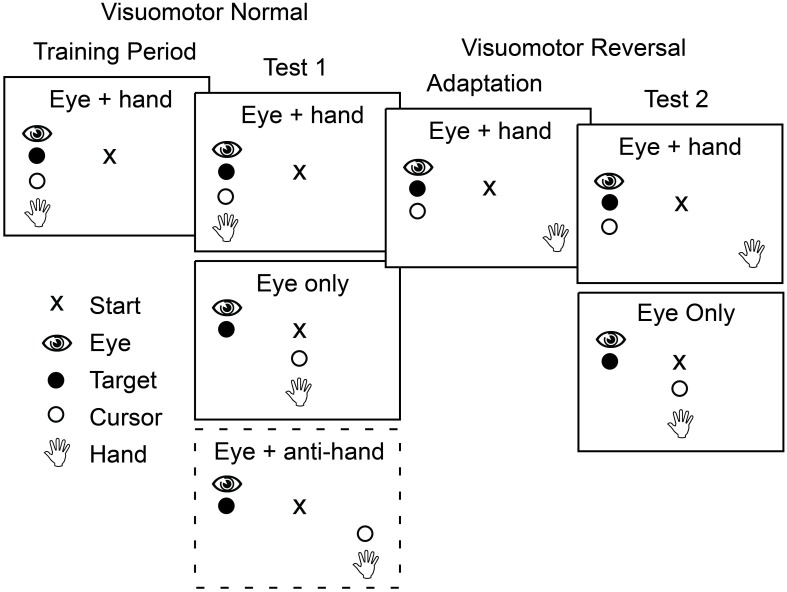
**Schematic outline of the four phases that participants completed in both Experiment 1 and Experiment 2.** In the first two phases, training and test 1, the mapping between position of the hand and the cursor controlled by the hand was veridical. In contrast, in the last two phases the cursor position was rotated 180° about the start position such that a rightward hand movement produced a leftward cursor movement. During training and adaptation, participants made only coordinated eye and cursor movements to the target. During the two test phases, participants made both eye only movements and eye plus cursor movements with these two trial types presented either in blocks (Experiment 1) or randomly interleaved (Experiment 2). In the first test phase in Experiment 1, participants also completed a block of trials in which they had to move the cursor away from the target while shifting gaze to the target (dashed box).

Figure 2 shows the sequence of experimental conditions. The experiment began with a training period in which participants moved the cursor, and therefore their gaze as well, to the presented target on each trial. Each target location was presented six times in a randomized order yielding 36 training trials.

After training, participants performed test trials presented in blocks based on movement type. There were three movement types. In *eye only* trials participants were instructed to look at the target without a hand movement. In *eye* + *hand* trials participants were instructed to move the cursor (and hence the hand) to the target. These cursor movements were always accompanied by an eye movement that shifted gaze to the target. In *eye* + *anti-hand* trials participants were instructed to look at the target but move the cursor (and hence the hand) away from the target in the opposing direction. Four trials for each of the six target locations were randomly ordered within each block and four such blocks of 24 trials were completed for each of the movement types for a total of 12 blocks. This yielded 288 trials in total with 16 trials for each combination of target location and movement type.

Following the first test period, participants were adapted to a visuomotor reversal where the direction of cursor movement was rotated 180° from the direction of hand movement. Thus, to move the cursor to the right, participants had to move their hand to the left and vice versa. During this adaptation period, participants completed 40 *eye* + *hand* trials per target location in randomized order (240 trials in total). Previous studies have shown that most participants can adapt to visuomotor rotations within 240 trials (e.g., Krakauer et al., 1999; Wigmore et al., 2002; Caithness et al., 2004).

After the adaptation trials, and with the visuomotor reversal in effect, participants completed a second test phase. Only two movement types were included in this phase of the experiment: *eye only* trials and *eye* + *hand* trials where participants were instructed to move the cursor to the target (requiring a hand movement in the direction opposite the target). With this instruction, participants always shifted their gaze to the target. There were four blocks of 24 trials for each movement type and the eight blocks, in total, were performed in a randomized order. Within each block, there were four trials for each target location randomly interleaved within each block. This yielded 192 trials in total with 16 trials for each combination of target location and movement type.

#### Experiment 2

The second experiment was similar in format to the first with the following major exception: Movement types were randomly interleaved over trials rather than blocked. The type of movement required on a trial was indicated by the color of the start position (red or blue) at each trial onset. For half the participants, blue indicated an *eye only* trial and red indicated and *eye* + *hand* trial; for the remaining participants, the color instructions were reversed.

Participants in Experiment 2 performed the same sequence of training, test, adaptation, and re-test trials used in Experiment 1 except *eye* + *anti-hand* trials were not included (see Figure 2). In the training period, they completed 12 *eye only* trials and 12 *eye* + *hand* trials (two replicates per target) with the 24 trials randomly interleaved. The test trials (both before and after adaptation) included 36 trials (six trials per target) for each movement type: *eye only* and *eye* + *hand*. The 72 trials were randomly interleaved. Participants completed 180 *eye* + *hand trials* during the adaptation period in which they moved by the cursor 30 times to each of the six targets presented in randomized order.

### Data analysis

Hand and gaze positions in the horizontal plane of the targets were sampled at 1000 Hz. This involved over-sampling the gaze data (recorded by the ISCAN system at 240 Hz). The x (left–right) and y (anterior–posterior) hand and gaze positions were digitally smoothed using a low-pass, dual-pass, fourth-order Butterworth filter with cut-off frequencies of 14 and 25 Hz, respectively. The ISCAN system smoothed the gaze data on-line with a 10-point moving average. To compensate for the temporal delay produced by this averaging, we time advanced the gaze signal by 19 ms, equal to one over the sampling rate (240) multiplied by (10-1)/2. To determine the start and end of eye and hand movements, the x and y gaze and hand positions were differentiated with respect to time to obtain velocities in the horizontal plane. The slope of the resultant of these velocity signals was then computed. A saccade began when the gaze velocity slope exceeded 15 m/s/s and ended when the slope during the deceleration phase exceeded −15 m/s/s. Hand movement start and end times were determined in the same way using thresholds of 0.5 m/s/s and −0.5 m/s/s, respectively. For each gaze and hand movement, we determined movement amplitude and the peak resultant velocity, which for simplicity, we will refer to as peak velocity. We visually inspected all data scoring to ensure that this algorithm worked successfully.

For analysis, we removed trials in which the first saccade undershot or overshot the center of the target by more than 2 cm (2.5° visual angle). This resulted in the exclusion of less than 5% of all trials. The great majority of hand movements also landed within 2 cm of the target center, even in the *eye* + *anti-hand* condition in Experiment 1 in which both the hand and cursor moved away from the target. We also excluded trials in which participants made hand direction errors (i.e., when the hand started to movement in the incorrect direction for a given condition). In Experiment 1, hand direction errors occurred in 14% of the trials and were primarily observed in the *eye* + *anti-hand* trials and the *eye* + *hand* trials under the reversal. In Experiment 2, errors occurred in only 3.6% of the trials despite the increased uncertainty due to the random interleaving of movement conditions. The absence of *eye* + *anti-hand* trials in Experiment 2 presumably contributed to the lower error rate but practice effects (from Experiment 1) may also have contributed.

In order to compare saccadic velocities across conditions, it is important to control for possible differences in saccadic amplitude because saccadic velocity increases with amplitude (Bahill et al., 1975; Fuchs et al., 1985). Therefore, for each participant and for each combination of target direction, amplitude, movement type, reversal, and experiment, we determined the predicted peak saccadic velocity that would be expected if the participant made a perfectly accurate eye movement to the target. This involved fitting a linear regression line relating peak saccadic velocity to saccadic amplitude to the individual trial data. The slope and intercept were then used to find the predicted peak saccadic velocity corresponding to the amplitude of the target. Snyder et al. (2002) used a similar approach to assess differences in saccadic velocity across conditions. Because hand velocity also scales with movement amplitude, we computed predicted peak hand velocities using the same method.

Repeated measures ANOVAs, based on participant averages across trials, were used to assess the effects of movement conditions, target amplitude, and target direction on various parameters of the eye and hand movements including maximum velocity and reaction time. The alpha level was set at 0.05.

## Results

**Figure 3:**
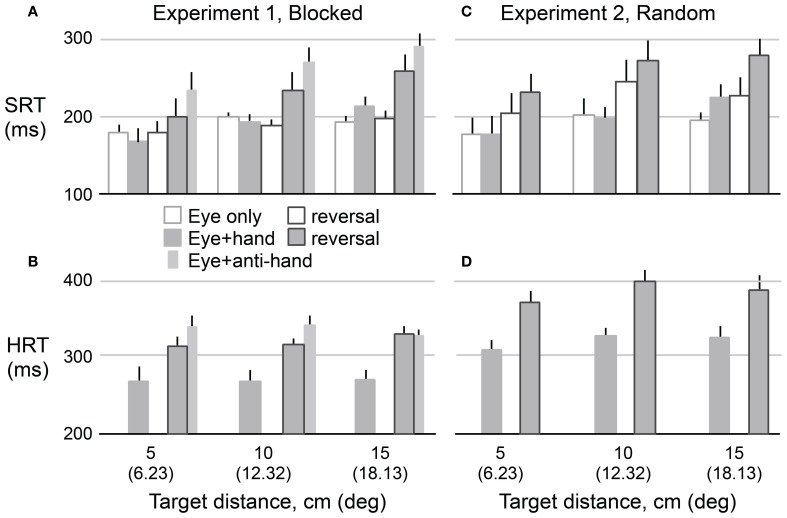
**Mean reaction time as a function of target amplitude for eye movements (SRT) in Experiment 1 (A) and Experiment 2 (C) and hand movements (HRT) for both experiments (B,D).** Hollow bars represent eye movement only conditions and filled bars represent eye plus hand movement conditions. The narrow bars represents mean reaction time for anti-hand movement trials. The vertical lines represent 1 SE.

Figure 3 shows means and standard errors (based on participant averages) of SRT **(A,C)** and HRT **(B,D)** as a function of movement type, visuomotor mapping (normal versus reversed), and target distance for Experiment 1 **(A,B)** and Experiment 2 **(C,D)**. The figure only includes data from the two test phases, and not from the adaptation phase. Because the results for leftward and rightward targets were very similar (as revealed by preliminary analyses of SRT, HRT, and saccadic velocity), the data were collapsed across target direction. In the following analysis, we excluded *eye* + *anti-hand* trials because they were only included in the Experiment 1 and only in the pre-adaptation phase. The results for this movement type are described at the end of the Results. As expected, One-Way repeated measures ANOVA revealed that HRT (Mean ± SE: 322 ± 7 ms) was greater (*P* < 0.001) than SRT (209 ± 8 ms). We used separate repeated measures ANOVAs to examine the effects of our experimental manipulations on HRT and SRT.

### Hand reaction time

A 2 × 2 × 3 repeated measures ANOVA was used to assess the effects of trial structure (i.e., blocked versus randomly interleaved *eye only* and *eye* + *hand* trials), visuomotor mapping, and target distance on HRT. As predicted, HRT was longer (*P* < 0.001) under the visuomotor reversal (350 ± 9 ms) than under normal visual feedback conditions (293 ± 7 ms). The trial structure also strongly affected HRT (*P* < 0.001). HRT was about 57 ms longer when movement types were randomly interleaved (350 ± 8 ms) compared to when they were delivered in blocks (292 ± 8 ms). HRT did not differ across target distances (*P* = 0.10) and target distance did not interact with other factors.

### Saccadic reaction time

A 2 × 2 × 2 × 3 (movement type, trial structure, visuomotor mapping, target distance) repeated measures ANOVA was used to examine experimental effects on SRT. Note that this analysis of SRT includes a factor (i.e., movement type) not included in the analysis of HRT, because HRT could not be computed for *eye only* trials. Unlike HRT, SRT increased with target distance (189 ± 13, 215 ± 8, and 222 ± 5 ms for the 5, 10, and 15 cm targets, respectively; *P* = 0.011). The increase in SRT with distance was larger for *eye* + *hand* trials compared to *eye only* trials resulting in an interaction between target distance and movement type (*P* = 0.001). SRT was longer (*P* = 0.007) for *eye* + *hand* trials (220 ± 9 ms) than for *eye only* trials (198 ± 8 ms). There was no reliable main effect of trial structure on SRT.

Our main research question concerned the effect of the visuomotor reversal on SRT. We were primarily interested in *eye* + *hand* trials but also asked whether SRT in *eye only* trials would be affected by adaptation to the visuomotor reversal. Overall, SRT was longer (*P* = 0.017) when visual feedback was reversed (225 ± 10 ms) compared when visual feedback was veridical (192 ± 8 ms). However, there was an interaction between movement type and visuomotor mapping (*P* = 0.031) with the effect of the visuomotor reversal being stronger for *eye* + *hand* trials than *eye only* trials. Therefore, we carried out separate 2 × 2 (trial structure, visuomotor mapping) repeated measures ANOVAs for each movement type. For *eye* + *hand* trials, there was a main effect of visuomotor mapping where SRT was clearly delayed (*P* = 0.024) under reversed (241 ± 15 ms) compared to veridical (194 ± 8 ms) visual feedback. However, there was no effect of trial structure and no interaction between trial structure and visuomotor mapping. For *eye only* trials, a main effect of visuomotor mapping was also observed where SRT was longer (*P* = 0.039) under reversed (206 ± 9 ms) compared to veridical (190 ± 7 ms) visual feedback. There was no main effect of trial structure. However, the interaction between trial structure and visuomotor mapping was marginally significant (*P* = 0.064). Further analysis revealed a reliable simple main effect of visuomotor mapping when trials were interleaved (*p* < 0.05) but not when trials were blocked.

### Relationship between hand and saccadic reaction times

As described above, in *eye* + *hand* trials the visuomotor reversal produced increases in both HRT, as expected, and SRT, as hypothesized. Moreover, the increases in HRT and SRT were roughly similar in magnitude. On average, HRT was 57 ms longer under the reversal whereas SRT, in the same *eye* + *hand* trials, was 50 ms longer under the reversal. To test whether the effects of the reversal on HRT and SRT were different, we carried out a 2 × 2 × 2 (effector, trial structure, visuomotor mapping) repeated measures ANOVA using *eye* + *hand* trials. No interaction between effector and visuomotor mapping was observed (*P* = 0.406) indicating that the reversal produced similar increases in HRT and SRT. No other two-way interactions were observed and there was no three-way interaction.

**Figure 4:**
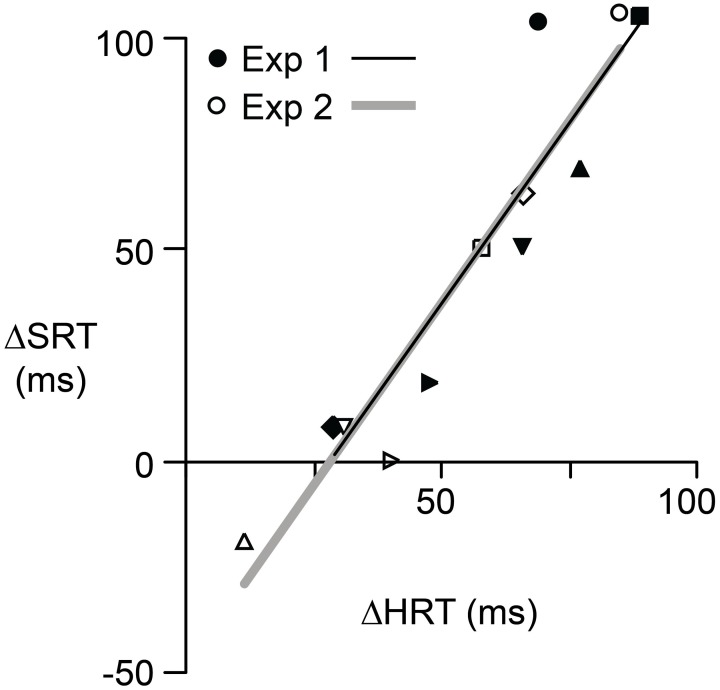
**The relationship between Δ SRT and Δ HRT for each participant in Experiment 1 (filled symbols) and Experiment 2 (open symbols).** Δ represents the effect of reversal adaptation, i.e., the difference between pre- and post-adaptation reaction time when the eyes and hand moved concurrently. A positive value indicates the reaction time was longer after adaptation. Different symbols are used for each of the six participants. Separate regression lines are shown for each experiment. However, these two lines have similar slopes and intercepts.

If the initiation of saccadic eye movements depends, in some way, on hand movement planning and control, then there should be a correlation, across participants, between the effects of the reversal on HRT and the effects of the reversal on SRT in the same *eye* + *hand* trials. Figure 4 shows the relationship between Δ SRT and Δ HRT where Δ refers to the difference between pre- and post-adaptation reaction time and positive values indicate longer reaction times following adaptation. Although the effects of the visuomotor reversal on reaction time could, for some participants, be quite different for the two experiments, in both experiments the relationship between Δ SRT and Δ HRT was linear and strongly positive (*r*^2^ = 0.88; *P* = 0.02 for Experiment 1 and *r*^2^ = 0.97; *P* = 0.001 for Experiment 2). For Experiment 1, the intercept and slope were −46.9 ms and 1.7 and for Experiment 2 the intercept and slope −47.9 ms and 1.7. The fact that the slope, in both cases, is greater than one suggests that the relative effects of the reversal on HRT and SRT varied with the effect on HRT. That is, the increases in HRT and SRT, due to the reversal, were most similar when the reversal produced larger increases in HRT.

### Peak saccadic and hand velocities

**Figure 5:**
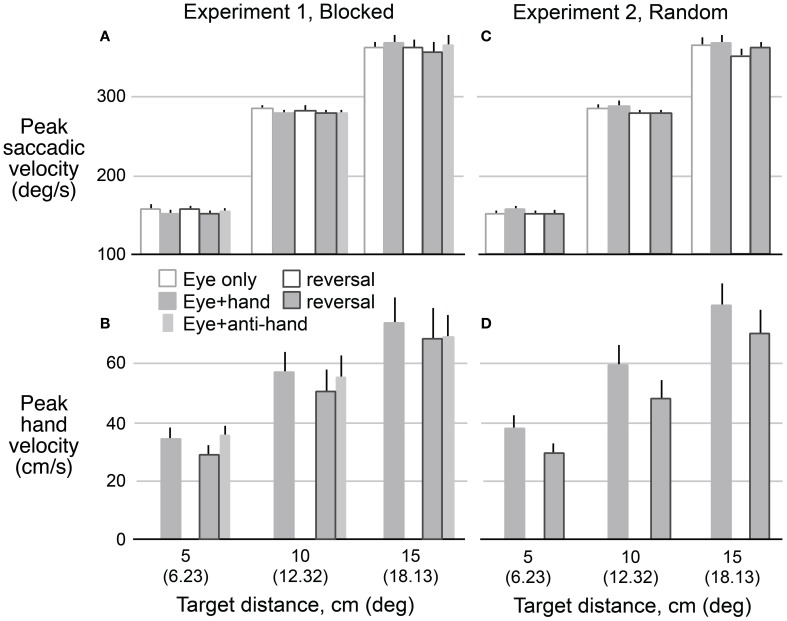
**Mean predicted velocity as a function of target amplitude for eye movements in Experiment 1 **(A)** and Experiment 2 **(C)** when eyes moved without hand movements (hollow bars) and with hand movements (filled bars) before and after the reversal adaptation.** Hand movements in both experiments **(B,D)** are also shown for pre- and post-adaptation. Anti-hand movement condition in Experiment 1 is shown by the narrow bars. The vertical lines represent 1 SE.

Figure 5 shows means and standard errors (based on participant averages) of saccadic **(A,C)** and hand **(B,D)** velocities (corrected for saccadic and hand movement amplitude, respectively, see Methods) as a function of target distance, movement type, and reversal for both Experiment 1 **(A,B)** and Experiment 2 **(C,D)**. As with reaction times, we collapsed across movement directions because the results for leftward and rightward targets were very similar. A 2 × 2 × 2 × 3 (trial structure, movement type, visuomotor mapping, target distance) repeated measures ANOVA revealed that saccadic velocity increased with target distance (*P* < 0.001) and was lower (*P* = 0.029) with reversed (265 ± 3°/s) compared to veridical (270 ± 3°/s) visual feedback. There was no main effect of trial structure or movement type but the two factors interacted (*P* = 0.014). Specifically, for blocked trials (Experiment 2), saccadic velocity was faster for *eye only* trials (269 ± 3°/s) than *eye* + *hand trials* (267 ± 4°/s) whereas, for randomly interleaved trials (Experiment 1), saccadic velocity was faster for *eye* + *hand* trials under blocked conditions but faster for the *eye* + *hand* trials (269 ± 4°/s) than eye only trials (266 ± 3°/s). The latter finding is consistent with the effect reported by Snyder et al. (2002) who randomly interleaved trials with and without hand movement.

A 2 × 2 × 3 (trial structure, movement type, target distance) repeated measures ANOVA confirmed that hand velocity increased with target distance (*P* < 0.001). Hand velocity was greater (*P* < 0.05) before adaptation to the visuomotor reversal (570 ± 52 cm/s) than after adaptation (493 ± 52 cm/s). Thus, although participants learned to make quite rapid hand movement under the reversal, hand speeds did not match those observed prior to the adaptation period. No effect of trial blocking was observed on hand velocity.

### Relationship between saccadic reaction time and saccadic velocity

Because SRT and saccadic velocity varied across movement conditions, the question arises whether there is a link between them. To examine this question we computed the correlation between SRT and saccadic velocity for each participant and each combination target distance, movement type, reversal, and trial structure. For each of these the 24 combinations (3 × 2 × 2 × 2), we computed the mean correlation coefficient averaged across participants. Correlations significantly different than zero were found for only 2 of the 24 cases and both of these were small (*r*^2^ < 0.198). Thus, we did not find evidence for a robust relationship between saccadic velocity and SRT. This finding is consistent with the results of Snyder et al. (2002) who found that the increase in saccadic velocity with a coordinated hand movement was independent of SRT.

### Hand and saccadic reaction times during adaptation

**Figure 6:**
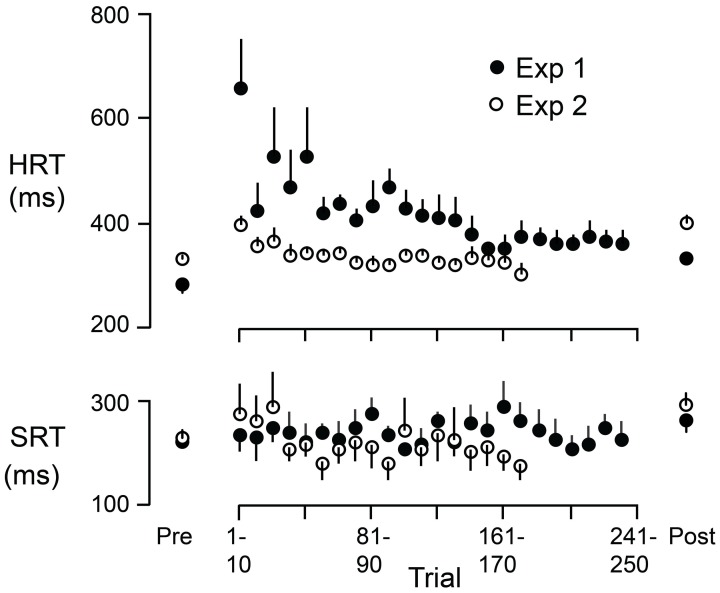
**HRT and SRT as a function of trial during the adaptation periods of Experiment 1 (filled circles) and Experiment 2 (open circles)**. Each point represents the mean across participants where the reaction time for each participant is the average across 10 successive trials. The figure also shows, for each experiment, HRT and SRT for eye + hand trials during the pre-adaptation and the post-adaptation phases. Each point represents the mean across participants. The vertical lines represent 1 SE.

Figure 6 shows HRT and SRT (means and standard errors based on participant averages) as a function of successive 10 trial blocks during the adaptation periods of Experiment 1 (filled circles) and Experiment 2 (open circles). The figure also shows, for each experiment, HRT and SRT for eye + hand trials during the pre-adaptation and the post-adaptation phases (means and standard errors based on participant averages). During the adaptation period of Experiment 1, experienced first by all participants, HRT decreased over the first 150 trials or so and then leveled off. At the start of the adaptation period of Experiment 2, HRT was reduced compared to the start of the same period in Experiment 1. HRT then decreased slightly before leveling off. Thus, as judged by HRT, participants appeared to retain learning of the visuomotor reversal across the successive experimental sessions. Because all participants completed Experiment 1 first, we cannot logically rule out the possibility that the reduced HRT at the start of the adaptation period in Experiment 2 (compared to Experiment 1) is due to some other factor other than learning across experiments. However, we would emphasize that participants only performed *eye* + *hand* movements during the adaptation phases of both Experiments and can think of no reason why adaptation of HRT would be different—apart from the learning or order effect. In any event, HRT at the end of the adaptation period was similar in the two experiments. This is consistent with the observation (see above) that the increase in HRT due to the reversal was similar in Experiments 1 and 2. Interestingly, the changes in HRT, within and across successive adaptation periods, are qualitatively similar to changes in direction error observed when participants adapt to a visuomotor rotation over successive sessions (e.g., Krakauer et al., 1999; Wigmore et al., 2002). Finally, in contrast to HRT, SRT did not appear to change appreciably during the adaptation period (Figure 6). We observed that early in the adaptation period, some participants occasionally kept their gaze at the start marker, presumably to watch which way the cursor would go, and only then shifted their gaze to the target. Although these trials were excluded from our analysis (due to excessive SRTs; see Methods), this gaze strategy resembles the gaze behavior observed when participants first learning a complex visuomotor transformation (Sailer et al., 2005).

### Hand and saccadic reaction times in anti-hand trials

Although we have not focused on *eye* + *anti-hand* trials, the results shown in Figures 3A,B indicate that HRT and SRT were prolonged in these trials even more than in *eye* + *hand* trials under the reversal. A One-Way repeated measures ANOVA, performed on the data from Experiment 1, confirmed that both HRT (*P* = 0.039) and SRT (*P* = 0.016) were greater in *eye* + *anti-hand* trials (HRT: 338 ± 10 ms; SRT: 274 ± 16 ms) than in *eye* + *hand* trials under the reversal (HRT: 318 ± 7 ms; SRT 231 ± 23 ms). This result provides additional evidence of a coupling between eye and hand movement initiation. The result also indicates that moving the hand away from the target is less disadvantageous when the cursor moves to the target than when it moves with the hand.

## Discussion

As expected, we found that the visuomotor reversal produced a marked increase on HRT. This effect on HRT, which was consistent across experimental sessions, permits us to scrutinize our main research question—whether an increase in HRT under the reversal would be accompanied by an increase in SRT. Our results, clearly answering this question in the affirmative, provide strong support for the hypothesis that processes involved in the programming of hand movement in visually guided reaching influence the initiation of eye movements directed to the reach targets.

In trials involving coordinated eye and hand movement, the reversal increased SRT by an average of 50 ms, an effect comparable to the 57 ms increase in HRT caused by the reversal. Critically, the increase in SRT occurred despite the fact that the required eye movement was unaffected by the visuomotor reversal. That is, participants always directed their gaze to the target. The rough equivalence between increases in HRT and SRT is consistent with the results of Fisk and Goodale (1985) showing that both hand and eye movements are delayed by some 405–0 ms when reaching to targets in contralateral versus ipsilateral space. In other words, Fisk and Goodale found that SRT for a given target varied depending on whether the reach was performed by the ipsilateral or contralateral hand. Importantly, for coordinated eye and hand movements, we found a strong coupling, across participants, between the effects of the reversal on HRT and SRT. In general, the greater the increase in HRT caused by the reversal, the greater the increase in SRT. This finding is consistent with previous work showing a correlation between HRT and SRT (Gielen et al., 1984; Mather and Fisk, 1985; Frens and Erkelens, 1991; Bekkering et al., 1995; Neggers and Bekkering, 1999, 2002).

Our results suggest that in rapid visually guided reaching to targets presented in peripheral vision, saccade initiation depends on, or is yoked to, hand movement. The question remains as to why saccade initiation would be delayed when hand movement initiation is delayed. Several studies of eye-hand coordination have suggested that hand movement commands are specified in gaze-centered coordinates (Andersen and Buneo, 2002; Engel et al., 2002; Crawford et al., 2004) and that both visual targets and the hand are represented in gaze-centered frames of reference in the posterior parietal cortex (Batista et al., 1999; Buneo et al., 2002; Medendorp et al., 2003; Khan et al., 2005), premotor cortex (Mushiake et al., 1997) and the superior colliculus (Stuphorn et al., 2000). One reason why saccade initiation may be delayed when additional time is required to initiate hand movement is because a saccade lunched too early may disrupt or distort the internal representation of the target before the hand movement is specified (Henriques and Crawford, 2000). Although this representation could be updated quickly during or even prior to the saccade (Duhamel et al., 1992; Jordan and Hershberger, 1994; Colby et al., 1995), even a brief disruption may be undesirable in a manual reaction time task such as the one we examined. This notion may also explain why SRTs increase when eye movements are accompanied by hand movement compared to when they are made in isolation (Mather and Fisk, 1985; Bekkering et al., 1994, 1995). Assuming that hand movement planning takes longer than eye movement planning (a reasonable assumption given that the geometry and dynamics of the arm are more complex than those of the eye), a delay in saccadic initiation would be expected. Interestingly, Bekkering et al. (1994) have shown that whereas SRT is delayed when eye movement is accompanied by a hand movement to the same target, SRT is not delayed when the hand is required to make a button press response. Because button pressing does not require specification of target location, there would be no need for the saccade to “wait for the hand”.

The delayed saccadic initiation observed under the visuomotor reversal may be compared to the gaze-locking mechanism reported by Neggers and Bekkering (2000, 2001) whereby participants, during rapid target pointing movements, failed to comply with the instruction to generate a saccade to a new visual target (presented during the reach) until the hand reaches the vicinity of the hand target. Neggers and Bekkering (2001) suggested that gaze is anchored on the reach target so that retinal and extraretinal gaze-related signals can be used to ensure pointing accuracy. Keeping gaze on target until the hand arrives may also maintain correlations between different sensory signals (e.g., visual, proprioceptive, tactile) and predicted sensory signals linked to goal achievement; correlations that would be used to uphold the sensorimotor mappings required for visually guided actions (Johansson et al., 2001). In contrast, we are suggesting that, in our task, gaze is “anchored” at the fixation point (until hand movement is specified) so as to ensure a stable reach target representation. Although the function of gaze may differ in the two situations, it is possible that similar pathways are involved in inhibiting gaze shifts based on hand movement signals.

The anchoring of gaze on the target during hand movement (Neggers and Bekkering, 2000, 2001) and the apparent anchoring of gaze prior to hand movement, observed in the present study, suggests that there must be a signal from brain circuits involved in hand movement planning and control to the circuits underlying saccade initiation. Neggers and Bekkering (2001) suggested that this linkage may involve the interaction between saccadic neurons in the superior colliculus (SC) and putative reach-related neurons in the same structure (Stuphorn et al., 2000) that are thought to receive projections from premotor and motor cortices (Fries, 1984, 1985; Werner et al., 1997a,b). However, there are also extensive interactions between gaze and hand movement related signals in parietal cortex (Colby et al., 1995; Crawford et al., 2004) and frontal cortex (Fujii et al., 2002).

The effect of the visuomotor reversal on SRT was not confined to coordinated eye and hand movements. When *eye* + *hand* and *eye only* trials were randomly interleaved (Experiment 2), the reversal led to an increase in SRT in *eye only* trials. In contrast, no such effect was observed when the different trial types were blocked (Experiment 1). The increase in SRT in randomly interleaved *eye only* trials can be explained if one assumes that hand movement are programmed in all trials, that the execution of the hand movement is actively inhibited in the *eye only* trials, and that the inhibition of the hand delays saccadic initiation. In contrast, when the two types of trials are delivered in separate blocks, participants presumably do not prepare hand movements in *eye only* trials and inhibition of hand execution is not required.

Snyder et al. (2002) showed that, in non-human primates, there is a small but reliable increase in saccadic velocity when simultaneously executed eye and hand movements are directed to the same target and not when the eye and hand movements is directed to different target located in opposite directions. We replicated the basic finding of Snyder and co-workers by showing that saccadic velocity increased for eye movement accompanied by a hand movement to the same target for trials with veridical visual feedback of hand movement in the experiment with randomly mixed *eye only* and *eye* + *hand* trials (Experiment 2). However, hand movement did not facilitate saccadic velocity when *eye only* and *eye* + *hand* trials were performed in blocks (Experiment 1). As suggested above, when these trial types are randomly mixed, hand movements may be actively inhibited during *eye only* trials. A spilling over of this putative hand movement inhibition to eye movement in the randomly mixed condition may account for the lower saccadic velocity in *eye only* trials compared to *eye* + *hand* trials. When the different trial types are delivered in blocks, hand movements need not be inhibited in *eye only* trials and hence no decrease in saccadic velocity is observed.

In previous studies showing facilitation of saccadic velocity by hand movement, the effect has been demonstrated under conditions in which there was spatial congruency of eye and hand movement directions as well as eye and hand targets (Epelboim et al., 1995; Snyder et al., 2002). We sought to determine whether spatial congruency of spatial targets alone could produce this phenomenon. To this end, we compared *eye only* and *eye* + *hand* trials performed under the visuomotor reversal in these trial types were randomly mixed. We found that saccadic velocity still tended to be greater in *eye* + *hand* trials but that the effect was marginally non-significant. Thus, whereas we can speculate that target congruency contributes to the hand effect on saccadic velocity, we cannot rule out a contribution of movement direction congruency.

Although it is well established that the coordination of eye and hand movements involves parietofrontal circuits, little is know about how these circuits would handle visuomotor transformations that alter the mapping between visual inputs and motor outputs. Barash (2003) suggested that “paradoxical” neurons, found in both the prefrontal (e.g., Funahashi et al., 1993) and parietal (e.g., Zhang and Barash, 2000, 2004) cortices, may play a key role in this regard. These neurons, which have been identified in the context of the memory-guided anti-saccade task, exhibit both visual and motor responses. However, what distinguishes them from other visual-motor neurons is that the visual and motor responses can be differentially classified based on temporal and spatial criteria. Thus, the motor response may be linked to the direction of the target whereas its visual response may be linked to the timing of target presentation. It is tempting to speculate that similar populations of neurons may play a part in the control of hand movement under altered visuomotor conditions such as those employed here.

In summary, we have provided strong evidence supporting the hypothesis that, in visually guided reaching, processes involved in hand movement programming affect the initiation of saccadic eye movements that are naturally directed to the reach targets. Our results also suggest that processes related to hand movement control can influence saccades in trials requiring eye movements alone (*eye only* trials) when such trials are randomly interleaved with trials requiring hand movement (*eye* + *hand* trials) but not when these trials types are performed in blocks. Thus, our findings point to a strong influence of task set on the control of eye movements.

### Conflict of interest statement

The authors declare that the research was conducted in the absence of any commercial or financial relationships that could be construed as a potential conflict of interest.
